# Supplementary material for: Pan-Cancer Profiling of Intron Retention and Its Clinical Significance in Diagnosis and Prognosis
Source: Cancers (Basel). 2023 Dec 1;15(23):5689. doi: 10.3390/cancers15235689 (PMC10705125; doi:10.3390/cancers15235689)
Supplement: Supplementary file 1 [file cancers-15-05689-s001.zip › cancers-2690134-supplementary/Supplementary figures.docx]

****Supplementary figures****





Supplementary Figure S1. RNA integrity check and RNA-seq coverage for representative intron retention events in three LAML samples.

(A) The median transcript integrity number (mTIN) is a proxy for RNA integrity, and mTIN larger than 70 indicates high RNA integrity^[1]^. Most LAML samples had high RNA integrity. (B) Gene body coverage plot for three representative LAML samples. (C-E) RNA-seq coverage of three IR genes in the three LAML samples.





Supplementary Figure S2. Number of patients with paired normal and tumor samples (A) and PCA of global IR (B) in 14 cancer types.





Supplementary Figure S3. Statistics and functional enrichment of differential intron retention events (DIRs) between tumor and adjacent normal samples.

(A) Boxplot of median IRratios of down or up-DIRs in normal (blue) or tumor (red) samples. (B) Number of DIRs that were specific to one cancer type or shared by different number of cancer types. (C) Number of differentially retained introns per DIR gene.

**

**

Supplementary Figure S4. Three cancer-related genes that were detected with DIRs.

(A) Increased IR of *LZTR1* was detected in 3 cancers: COAD, STAD and UCEC. Left panel is boxplots of IRratio and mRNA expression (TPM) of each patient-matched normal and tumor samples. NS, not significant; *, *P* < 0.05; ***, *P* < 0.001; paired Wilcoxon test. Right panel shows a patient-matched normal and tumor sample RNA-seq read coverage from the respective cancer type. Patient IDs are shown besides each cancer type. (B) Increased IR of *ERCC4* was detected in 3 cancers: COAD, STAD and UCEC. (C) Decreased IR of *CSF3R* was detected in BRCA.


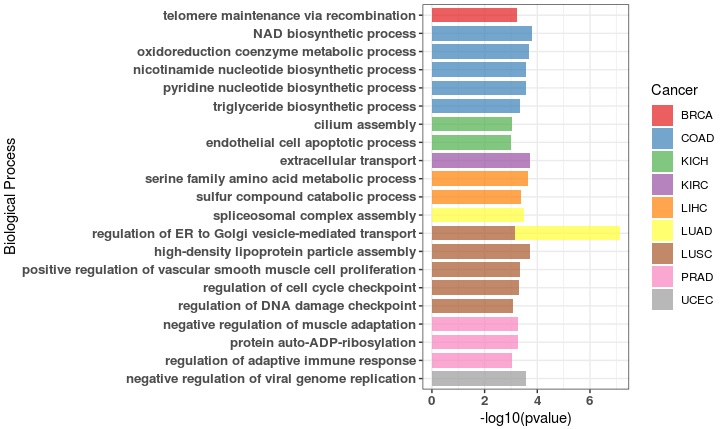


Supplementary Figure S5. DIR genes from different cancers were enriched in different GO terms.

**

**

Supplementary Figure S6. Percentage of introns predicted to cause nonsense-mediated decay (NMD) (A) and sequence conservation around intron boundaries (B) in four classes of introns.

**

**

Supplementary Figure S7. Comparison of 5′ splice signal (A), 3′ splice signal (B), intron GC content (C) and intron length (D) of up and down-regulated DIRs across 14 cancer types.

Dashed lines represent median values of constitutive introns (n = 48,344). Unlabeled, not significant; *, *P* < 0.05; ***, *P* < 0.001; Wilcoxon rank-sum test.





Supplementary FIGURE S8. Visualization of global and differential IR events in 14 cancers.

(A, B) tSNE plot of all introns colored by cancer types or by tumor or normal sample (n = 37,845). (C) tSNE plot of differential introns colored by cancer types or by tumor or normal sample (n = 375).



 Supplementary Figure S9. Random forest model in each type of cancer.

(A) For each cancer, respective DIRs with missing rate less than 30% were selected to build the Random Forests model, and paired tumor and normal samples were randomly divided for model training (75%) and testing (25%). (B) Number of DIR passing missing rate selection for each cancer. (C) AUC of random forest model in each cancer type.


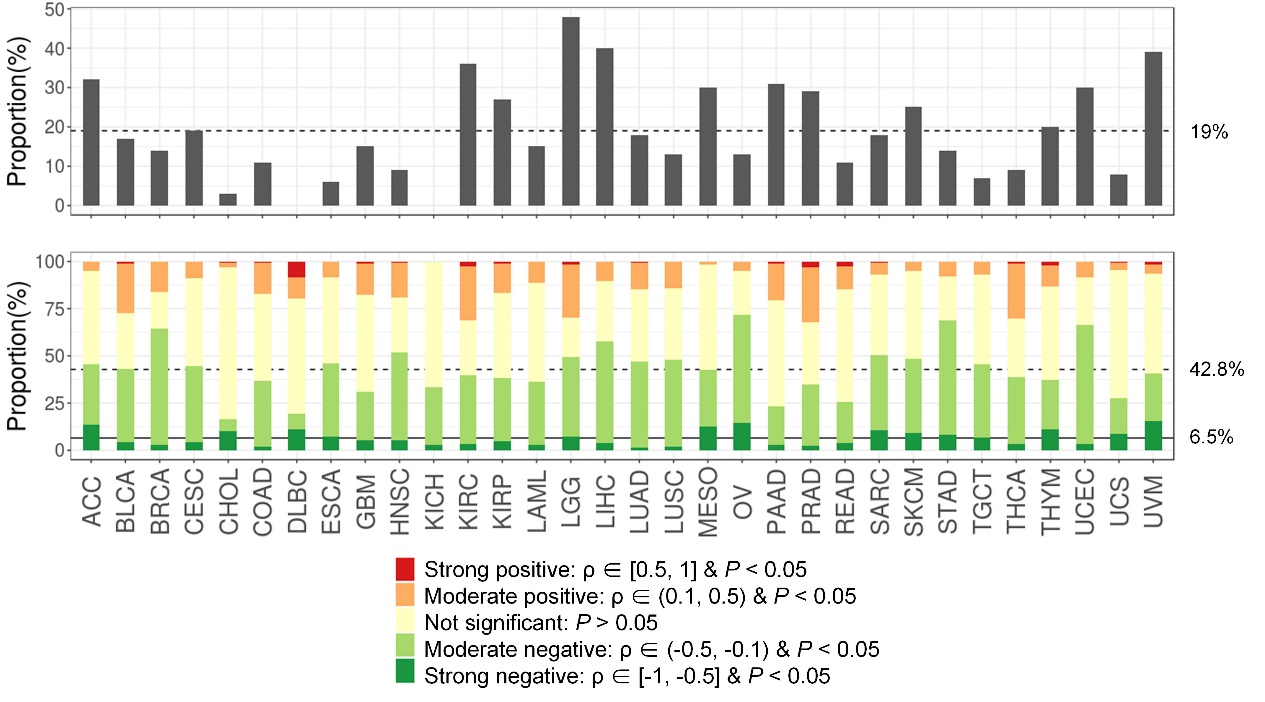


Supplementary Figure S10. Prognostic introns and related gene expression.

Top panel shows the percentage of prognostic IR genes whose expression was also associated with prognosis. The dotted line is the median value across cancer types. Bottom panel shows the correlation of IRratio and gene expression (TPM) in prognostic introns across cancers. Spearman correlation coefficients fall into six categories: strong positive, moderate positive, weak positive, weak negative, moderate negative and strong negative. The dotted line and solid line are the median proportions of all negative and strong negative correlations, respectively.





Supplementary Figure S11. Prognostic IR genes were enriched in cancer related KEGG pathways for many cancer types.

Cell color indicates *P* values (hypergeometric test; white indicates *P* values larger than 0.01).


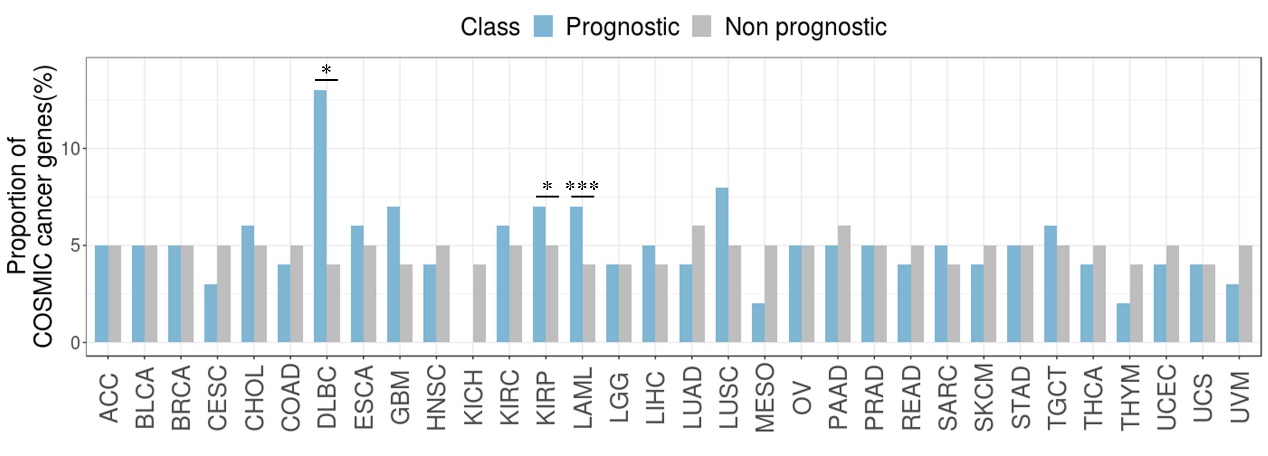


Supplementary Figure S12. Percentage of COSMIC cancer genes among prognosis IR and non-prognosis IR-related genes across different cancer types.

*, *P*<0.05; ***, *P*<0.001; Fisher’s exact test. The unlabled is not significant.


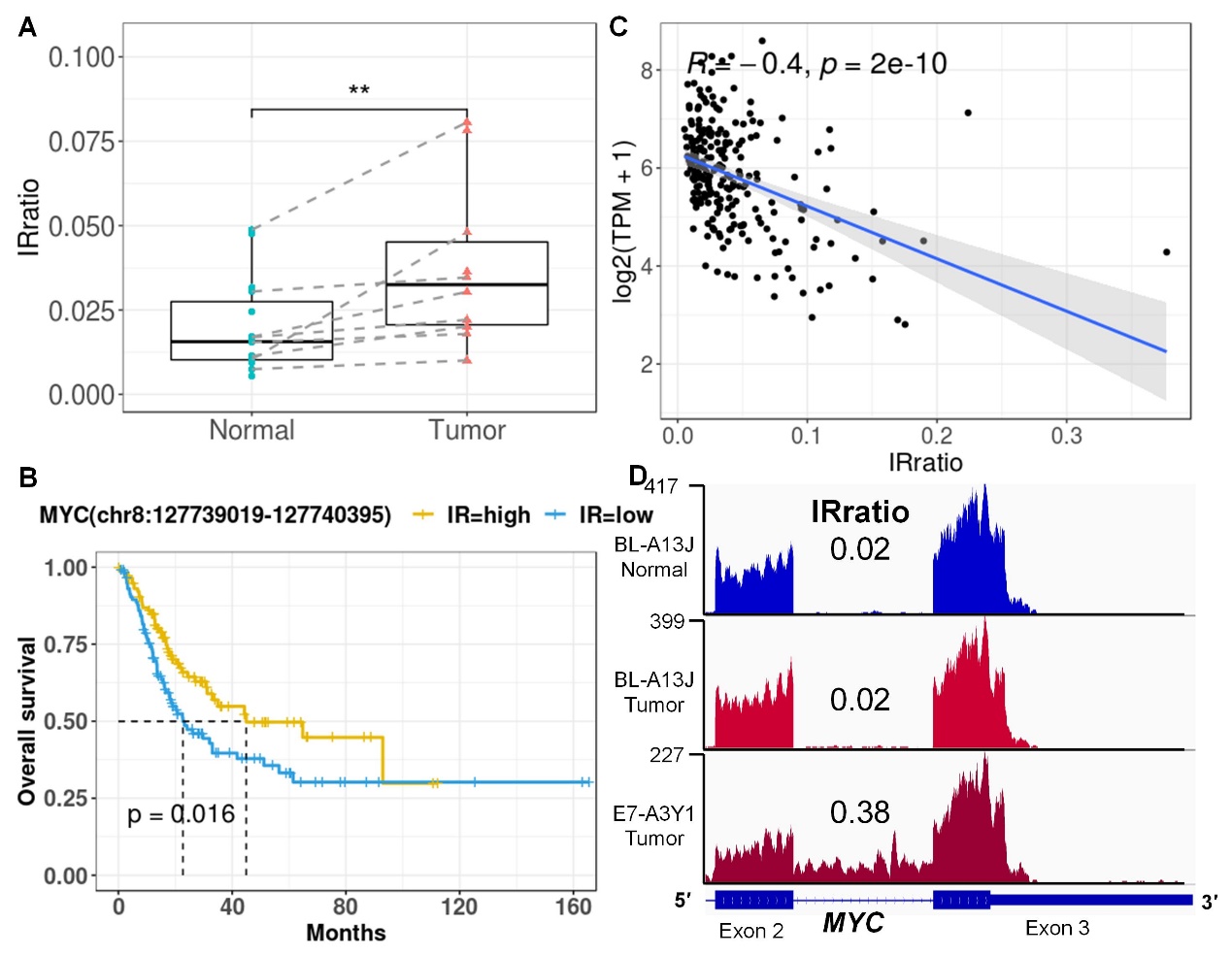


Supplementary Figure S13. The last intron of *MYC* is retained in a higher level in tumor samples, and is negatively correlated with gene expression and associated with prognosis in BLCA.

(A) The intron was differentially retained in matched tumor and normal samples. (B) Increased retention level was associated with favorable prognosis in BLCA patients. (C) Retention level negatively correlated with *MYC* expression in tumor samples. (D) RNA-seq read coverage from a matched normal and tumor sample and another tumor sample.


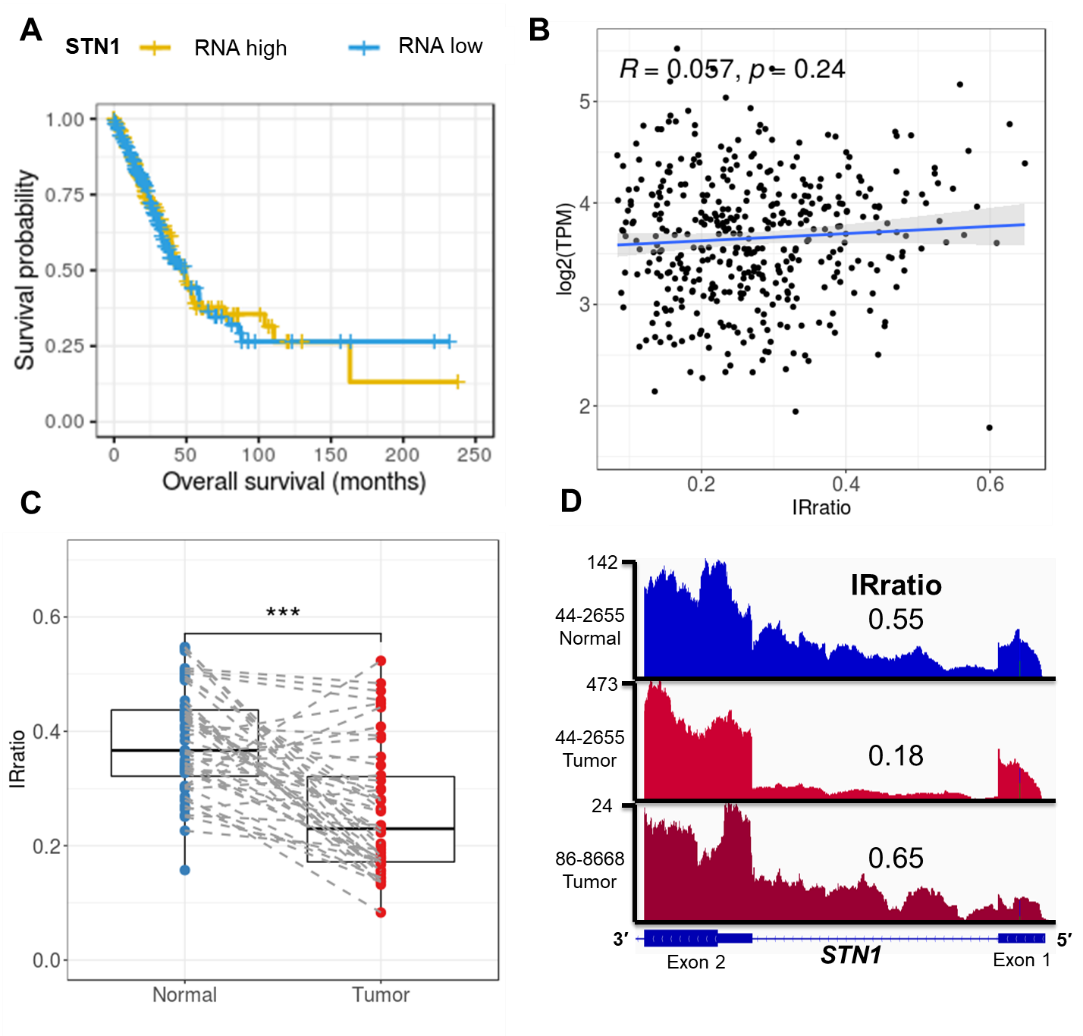


Supplementary Figure S14. RNA level of *STN1* is not associated with prognosis in LUAD, while the intron within its 5′UTR is not correlated with RNA level, and is retained in a lower level in tumor samples.

(A) RNA level of *STN1* is not associated with prognosis in LUAD patients. (B) Retention level of 5′ UTR intron is not correlated with *STN1* expression in tumor samples. (C) The intron is retained in a lower level in tumor samples. (D) RNA-seq read coverage from a matched normal and tumor sample and another tumor sample.

**
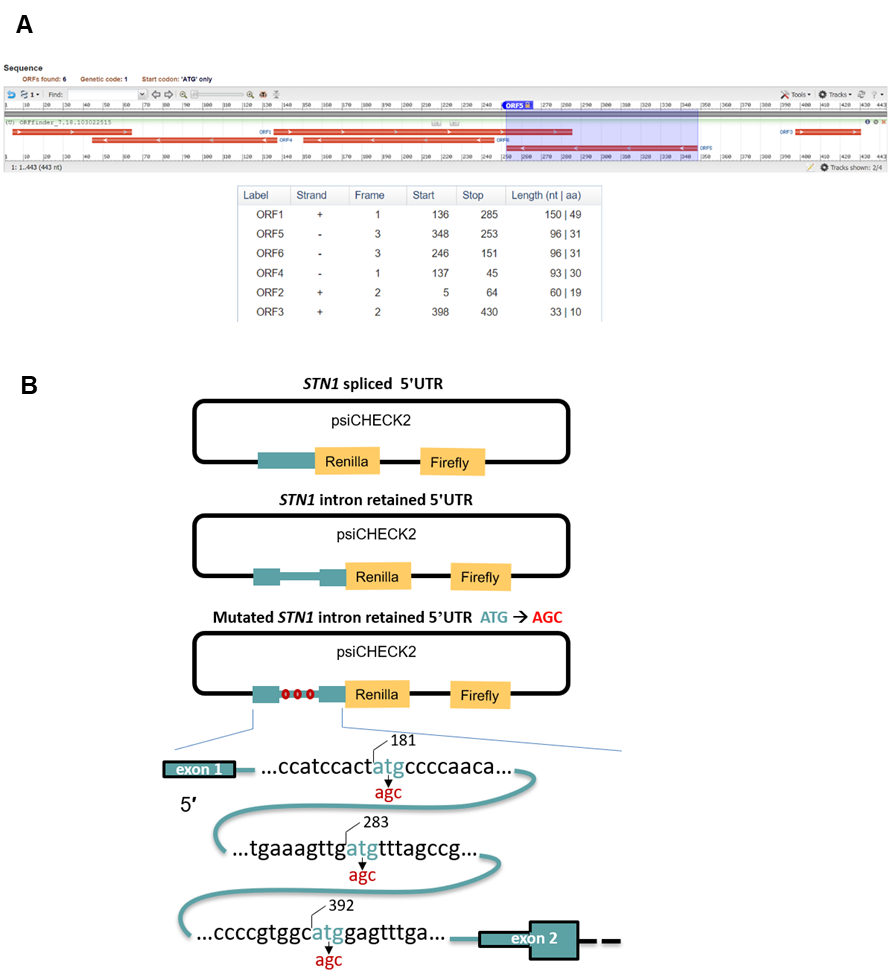
**

**Supplementary Figure S15. The intronic sequence of *STN1* 5′ UTR encodes three potential upstream open reading frames (uORFs).**

(A) uORFs predicted by ORFinder (<https://www.ncbi.nlm.nih.gov/orffinder/>). Because *STN1* is located on the minus strand, ORF4, ORF5 and ORF6 are three potential uORFs with lengths ranging from 93 to 96 nt. (B) Schematic diagram illustrating the design of the dual-luciferase reporter vector, where 3 different 5′ UTRs of *STN1* (spliced 5′ UTR, IR 5′ UTR, and mutated IR 5′ UTR (ATG to AGC)) were attached to the psi-CHECK2 vector, located in front of Renilla. We used psi-CHECK2 plasmids with three different STN1 5′ UTR sequences inserted upstream of the Renilla coding region: the spliced 5′ UTR (shorter), the intron-retained 5′ UTR (longer), and the intron-retained 5′ UTR with start codon ATGs mutated to AGCs (longer and mutated)

**
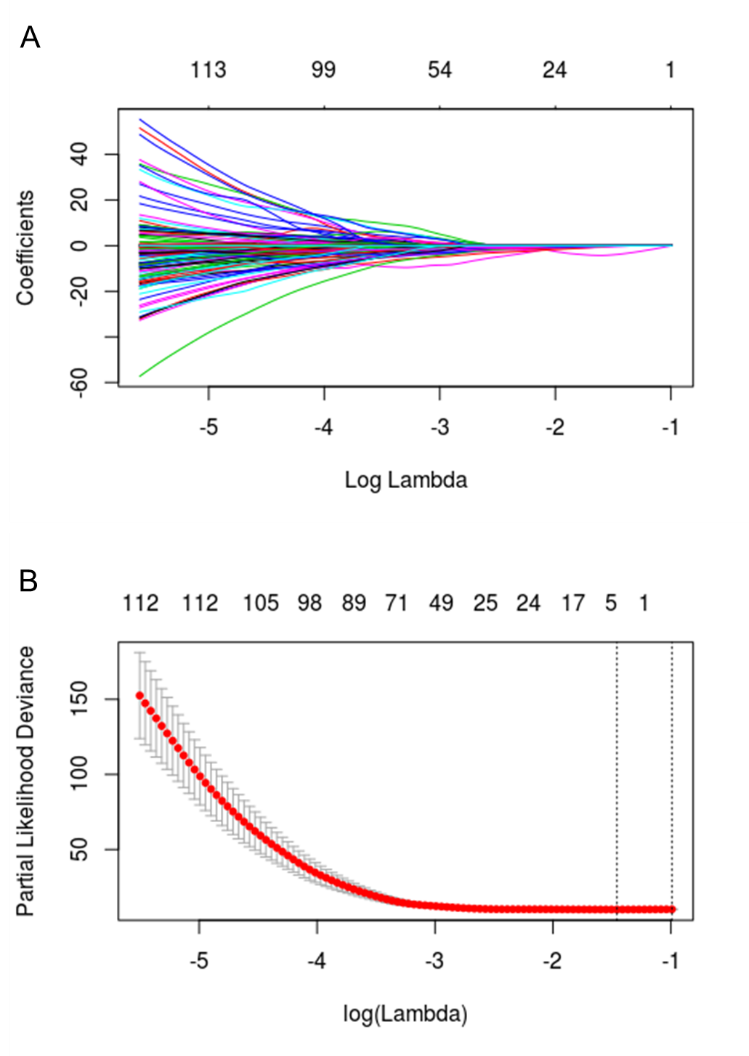
**

**Supplementary Figure S16. Four introns were selected to build a prognostic model for LAM L patients by LASSO regression.**

(A) Each curve represents the trajectory of the coefficients of each independent variable, with the vertical coordinate being the value of the coefficient, the lower horizontal coordinate being log(λ), and the upper horizontal coordinate being the number of non-zero coefficients at this point in the model. As the value of λ changes, the later the coefficient is compressed to zero the more important the variable is. (B) The value of λ controls the number of non-zero parameters of the model, as shown in the upper horizontal coordinate. Cross-validation yields confidence intervals for partial likelihood deviance corresponding to each λ value. There are two dashed lines in the figure, from left to right, corresponding to log(λ) when partial likelihood deviance is minimized and when the number of features is minimized, respectively.
